# Clinical therapeutic effects of topical agents in adult patients with human immunodeficiency virus-related oral mucosa ulcers: A protocol for systematic review and meta-analysis

**DOI:** 10.1097/MD.0000000000023626

**Published:** 2021-02-12

**Authors:** Xiuming Ding, Feihu Xu, Xiang Zhang

**Affiliations:** Department of Stomatology, Hai’an People's Hospital, Hai’an, Jiangsu Province, P. R. China.

**Keywords:** human immunodeficiency virus, oral mucosa ulcers, protocol, systematic review, topical agents

## Abstract

**Background:**

The number of adult patients affected by the human immunodeficiency virus (HIV) still remains high, mainly in the developing countries. However, only a few affected patients fail to experience oral lesions in the course of their experience with the virus. In particular, oral mucosa ulcers detected among HIV patients may be severe, which depictions may inhibit oral functioning and change patients’ quality of life. Thus, it can result in considerable morbidity among this group of patients. To this end, the present study aims to examine the topical agent's clinical therapeutic efficacy among adult patients suffering from HIV-related oral mucosa ulcers.

**Methods:**

For the investigation, only randomized controlled trials on any topical agent used to treat adult patients with HIV oral mucosa ulcers are to be explored from different databases: PubMed, the Cochrane Library, PsycINFO, EMBASE, SCOPUS, Web of Science, China Biomedical Literature Database, China National Knowledge Infrastructure, VIP, and WanFang databases. All databases will be searched from their inceptions to October 2020. Additionally, 2 independent authors will evaluate the possibly eligible studies to be included in the study. They will also perform data's trial extraction and risk of bias assessment. Accordingly, all data will be analysed by means of the RevMan 5.3 software.

**Results:**

The present study seeks to evaluate the topical agents’ clinical therapeutic efficacy to treat adult patients with HIV-related oral mucosa ulcers.

**Conclusion:**

The study can be applicable in providing evidence of any topical agents for treating adult patients with HIV-related oral mucosa ulcers for clinical practice.

**Protocol registration number:**

DOI 10.17605/OSF.IO/5CYR2 (https://osf.io/5cyr2/).

## 1 Introduction

Based on recorded data of the Joint United Nations Programme on human immunodeficiency virus (HIV)/acquired immunodeficiency syndrome (AIDS) (UNAIDS) of 2018, approximately 37.9 million people are HIV-positive, and nearly 770,000 deaths have been recorded from HIV-related origins, with about 1.7 million people getting newly infected with HIV.^[1]^ Similarly, almost all infected persons tend to develop an AIDS.^[2,3]^ Besides, the disease reveals itself in various adaptable illnesses and cancers in formerly well people, including Mycobacterium tuberculosis, Kaposi sarcoma, and pneumonia, among other illnesses.^[4–6]^ Additionally, patients experiencing AIDS tend to showcase weakened immune response, such as low lymphocyte proliferative responses ex vivo as well as exhaustion of T-helper cells.^[4–6]^ In essence, HIV infects CD4+ T cell and macrophages selectively while depleting CD4+ T cells, which is consistent with the onset of AIDS symptoms.^[7–10]^ In adults infected with HIV, oral mucosa ulcers are disposed to last longer in most cases, and generate more agonizing indications than experienced in immunocompetent individuals.^[11]^ Numerous studies have previously indicated that nearly 40% to 50% of HIV-positive individuals have oral fungal, bacterial, or viral illnesses, which usually transpire prematurely in the illness.^[12]^ Thus, the present study aims to perform an evaluation of the clinical therapeutic efficacy of topical agents with HIV-related oral mucosa ulcers.

## 2 Methods

### 2.1 Study registration

The protocol was registered on the Open Science Framework (OSF) (https://osf.io) (registration DOI number: 10.17605/OSF.IO/5CYR2). Besides, the protocol was composed in accordance with the Preferred Reporting Items for Systematic Reviews and Meta-Analyses Protocols statement guidelines.^[13]^

### 2.2 Criteria for study selection

#### 2.2.1 Types of studies

All available randomized controlled trials (RCTs) on any topical agent to treat adult patients with HIV oral mucosa ulcers in HIV positive patients will be included in the study. However, case reports, retrospective investigations, animal trials, evaluations, or non-RCTs will not be considered.

#### 2.2.2 Types of participants

HIV infected adults (above 18 years of age) with 1 or more intraoral mucosa ulcers.

#### 2.2.3 Types of interventions

The study's purpose is to examine the efficacy of topical agents’ clinical therapeutic in adult patients with HIV-related oral mucosa ulcers. We will treat intervention group with any topical agents, such as topical thalidomide, topical corticosteroids, antiseptic mouth washes (chlorhexidline, Cetylpyridinium Chloride Gargle), and any highly active antiretroviral therapy regimen plus any topical agent treatment.

#### 2.2.4 Types of comparisons

Furthermore, we will treat comparison group with any other topical agents or placebo, such as topical thalidomide, topical corticosteroids, antiseptic mouth washes (chlorhexidline, cetylpyridinium chloride gargle), and any highly active antiretroviral therapy regimen plus any topical agent treatment.

#### 2.2.5 Types of outcomes

The types of anticipated outcomes include pain relief, healing of ulceration, incidence after healing of ulcers, recurrent after healing of ulcers, duration to heal oral mucosa ulcerations subsequent to commencement of treatment, and adverse effects.

### 2.3 Search methods for identification of studies

#### 2.3.1 Electronic searches

These electronic databases will be explored: PubMed, the Cochrane Library, PsycINFO, EMBASE, SCOPUS, Web of Science, China Biomedical Literature Database, China National Knowledge Infrastructure, VIP, and WanFang databases. All databases will be explored from their inceptions to October 2020. Further, we will build a search strategy using the following mixing of keywords and free words: “HIV,” “oral mucosa ulcers,” “adult,” “randomised controlled trials.”

#### 2.3.2 Searching other sources

We will further explore reference lists of the included investigations, including the International AIDS Conference, the British HIV Association Conference, and the International Congress on Drug Therapy in HIV infection to identify additional trials.

### 2.4 Data collection and analysis

#### 2.4.1 Selection of studies

**Figure 1:**
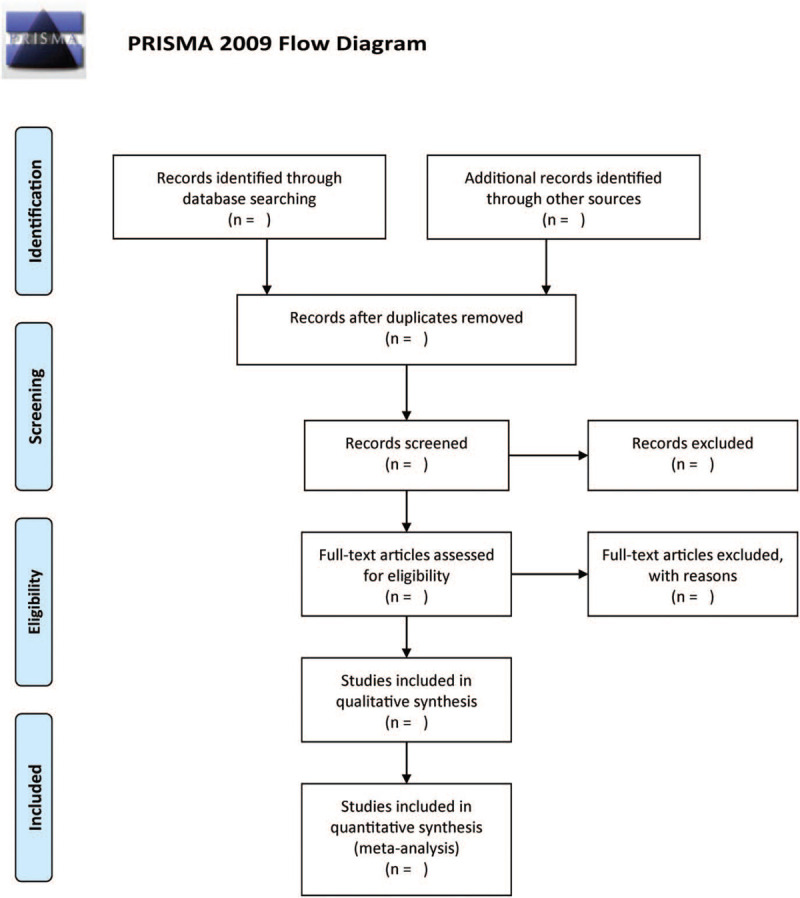
The flow diagram of the study selection process.

Two independent authors will examine the potential eligible studies for inclusion. After removing duplicates, the authors will use the full-text of the included investigations to assess which of them will finally be included in this study. Any disagreement will be addressed through deliberations or by consulting a third author. Figure 1 displays the flow chart.

#### 2.4.2 Data extraction and management

Two independent authors will utilize a standardized data extraction form to obtain relevant data from the included studies. They will extract information such as: first author, publication date, design, sample size, duration, intervention and comparison method, results, and adverse effects. Any disagreement will be addressed by deliberation, or, where possible, by consultation by a third author.

#### 2.4.3 Assessment of risk of bias

The study will employ the Cochrane Handbook to evaluate the risk of bias of each included RCT in the bass of the following domains: random sequence generation, allocation concealment, blinding, incomplete outcome data, and selective outcome reporting, among other source biases. The risks will be identified as “high risk,” “low risk,” or “unclear.” Any disagreement will be addressed by deliberation, or, where possible, by consultation by a third author.

#### 2.4.4 Measures of treatment effect

The pooled effects of continuous outcomes will be presented as risk ratio with 95% confidence intervals. The pooled effects of dichotomous outcomes will be presented as mean differences or standardized mean differences with 95% confidence intervals.

#### 2.4.5 Dealing with missing data

For trials with missing data, we will contact respective authors to seek clarification and supplementation of the data.

#### 2.4.6 Assessment of heterogeneity

We will use a total of 12 statistic to approximate statistical heterogeneity. We will more than 50% to denote that there is a significant statistical heterogeneity, thus we will utilize the random-effects model to analyze the pooled data^[14]^; If not, we will employ apply the fixed-effects model.^[15]^

#### 2.4.7 Assessment of reporting bias

Where over 10 studies have been used, we will plan to use funnel plots to evaluate the potential publication bias.

#### 2.4.8 Sensitivity analysis

To test the stability of our findings, sensitivity analyses will be conducted based on investigations considered to be of low risk of bias.

## 3 Discussion

Although published studies have reported that topical agents have been critical to treating patients with HIV-related oral mucosa ulcers in adults, the findings are still controversial. To date, no prior systematic review has been applied in evaluating the clinical therapeutic efficacy of topical agents in adult patients with HIV-related oral mucosa ulcers. Thus, we conduct the present study to examine systematically the clinical therapeutic efficacy of topical agents in adult patients with HIV-related oral mucosa ulcers. Our findings will provide valuable recommendations for the clinical treatment in patients with HIV-related oral mucosa ulcers in adults.

## Author contributions

**Conceptualization:** Xiuming Ding, Xiang Zhang.

**Data curation:** Xiuming Ding, Xiang Zhang.

**Formal analysis:** Feihu Xu, Xiang Zhang.

**Funding acquisition:** Feihu Xu.

**Investigation:** Feihu Xu.

**Project administration:** Feihu Xu.

**Resources:** Feihu Xu.

**Software:** Xiuming Ding, Feihu Xu, Xiang Zhang.

**Validation:** Feihu Xu, Xiang Zhang.

**Visualization:** Xiuming Ding.

**Writing – original draft:** Xiuming Ding, Xiang Zhang.

**Writing – review & editing:** Xiuming Ding, Xiang Zhang.

## Abbreviations

Abbreviations: HIV = human immunodeficiency virus, RCT = randomized controlled trials.
